# Disease site as an independent predictor of survival in radioiodine-refractory thyroid cancer

**DOI:** 10.3389/fendo.2026.1824512

**Published:** 2026-06-11

**Authors:** Giulia Sapuppo, Marco Russo, Grete Francesca Privitera, Michele Massimino, Sebastiano Piana, Maria Antonietta Trovato, Giusi Blanco, Davide Adinolfi, Giorgia Romano, Gabriella Pellegriti

**Affiliations:** 1 Endocrinology Section, Department of Clinical and Experimental Medicine, University of Catania, Garibaldi-Nesima Hospital, Catania, Italy; 2 Medical Oncology Unit, Istituto Clinico Humanitas, Catania, Italy; 3 Bioinformatics Unit, Department of Clinical and Experimental Medicine, University of Catania, Catania, Italy; 4 Department of General Surgery and Medical-Surgical Specialties, University of Catania, Catania, Italy; 5 Center of Experimental Oncology and Hematology, A.O.U. Policlinico “G. Rodolico-S. Marco”, Catania, Italy; 6 Diagnostic and Interventional Radiology Unit National Relevance and Highly Specialized Hospital (ARNAS) Garibaldi - Nesima, Catania, Italy; 7 Surgical Oncology, Garibaldi-Nesima Medical Center, Catania, Italy; 8 Oncology Unit, Garibaldi-Nesima Hospital, Catania, Italy; 9 Medical Oncology, Department of Clinical and Experimental Medicine, University of Catania, Catania, Italy

**Keywords:** advanced thyroid cancer, distant metastases, locoregional disease, outcomes, radioiodine-refractory thyroid cancer, real-world data, tyrosine kinase inhibitors (TKIs)

## Abstract

**Background:**

Differentiated advanced thyroid carcinoma (DATC) is rare, accounting for ~5% of thyroid tumors, including radioiodine-refractory thyroid tumors (RAI-R-TC) and thyroid tumors not amenable to thyroidectomy. Prognosis is poor, with a 10-year survival of ~10% and median life expectancy of 3–5 years. Systemic therapies are often required, but management remains challenging due to comorbidities and treatment-related toxicity. In particular, it is unknown whether outcomes differ between patients with predominantly locoregional disease and those with distant metastases.

**Patients and methods:**

We retrospectively analyzed a consecutive series of 43 patients with RAI-R-TC treated with tyrosine kinase inhibitors (TKIs). Patients were stratified according to the predominant site of disease involvement at TKI initiation: (i) locoregional progression with involvement of critical cervical structures, and (ii) progressive distant metastatic disease. Tumor response and disease progression were defined according to RECIST 1.1 criteria. Disease-specific survival (DSS) was estimated using the Kaplan-Meier method, and differences between the two groups were assessed using the log-rank test.

**Results:**

Median age at diagnosis was 63.3 years; 51.2% patients were females. Histology included papillary (n=26), follicular (n=10), poorly differentiated (n=6), and papillary–follicular (n=1). At presentation or shortly after, 44.2% patients had distant metastases; 55.8% had locally advanced disease, of whom 91.7% also developed distant spread. Patients with locoregional disease demonstrated significantly worse disease-specific survival compared with those presenting with distant metastases (p < 0.05). At last follow-up, 51.2% were stable, 11.6% progressed, 62.8% were alive, and 37.2% had died of disease. The difference in survival between the two groups was statistically significant.

**Conclusion:**

Despite belonging to the same disease entity, our preliminary data suggest that DATC patients show divergent prognostic outcomes depending on disease extent. Locoregional invasion appears to carry a particularly poor outcome, underscoring the need for differentiated and personalized therapeutic strategies. TKIs remain effective in both settings, but tailored management is warranted to address the distinct challenges of locoregional versus distant metastatic disease.

## 1 Introduction

Differentiated advanced thyroid carcinoma (DATC) is a rare condition, accounting for 5% of all thyroid tumors, with an incidence of approximately 4–5 cases per 1,000,000. DATC includes radioiodine-refractory thyroid carcinomas (RAI-R TC) and thyroid tumors not amenable to thyroidectomy, for which treatment with ¹³¹Iodine (¹³¹I) is not indicated. Approximately 60–70% of patients with distant metastases, representing about 5% of all thyroid cancer cases, are refractory to radioiodine, either ab initio or during follow-up, with a significantly negative impact on prognosis. These patients present a 10-year survival rate of approximately 10% and a median life expectancy of 3–5 years from diagnosis (1–5).

Patients with progressive DATC, in the absence of locoregional treatments to control the disease, are eligible for systemic therapies (5). In recent years, genomic profiling has enabled the development of targeted molecular therapies addressing key oncogenic driver mutations. This has significantly advanced the treatment landscape for thyroid cancer. An additional innovation is the recent “agnostic” approval of drugs whose therapeutic indication is independent of the primary tumor site and histology, relying solely on the presence of specific molecular alterations. This “histology-independent” characteristic underpins the concept of basket trials and supports the indication for treatment with specific inhibitory agents.

Tyrosine kinase inhibitors (TKIs), such as lenvatinib, sorafenib, and cabozantinib, as well as selective targeted inhibitors, such as selpercatinib, larotrectinib, entrectinib and the combination of dabrafenib and trametinib for BRAF V600E-mutated cases are indicated for the treatment of DATC. Multiple randomized controlled trials have shown that lenvatinib effectively improves progression-free survival (PFS), with a median PFS of 18.3 months, significantly longer than the 3.6 months observed in the placebo arm (6–9).

Other multi-kinase inhibitors, such as sorafenib and cabozantinib, are indicated in cases of toxicity or progression in patients treated with lenvatinib. Selpercatinib, recently approved, is indicated as monotherapy for RET fusion–positive DATC requiring systemic therapy, either as first-line treatment or after prior lenvatinib exposure. It is a selective RET inhibitor, and its efficacy was demonstrated in the LIBRETTO-001 phase I–II study, which reported a high overall response rate (ORR) of 79% (median duration 18.4 months) and a median PFS of 20.1 months (10).

Larotrectinib and entrectinib have also been approved for tumors with NTRK gene fusions, with an ORR of approximately 80% and a median PFS of 28.3 months.

The management of these rare conditions remains a clinical challenge, particularly in the presence of comorbidities, unresectable tumors, or therapy-related side effects.

To date, no studies have clearly demonstrated whether prognosis differs according to the predominant site of disease involvement. In clinical practice, DATC may manifest either predominantly, or even exclusively, with distant metastases, or instead with locoregional disease characterized by extensive involvement of critical cervical structures such as the trachea, esophagus, and major vessels. These two disease patterns can substantially influence not only the likelihood of response to systemic therapy, but also therapeutic decision-making and, ultimately, patient prognosis. Progressive locoregional disease, in particular, often necessitates palliative interventions and, in some cases, highly morbid procedures such as tracheostomy or laryngectomy.

This study aimed to characterize the clinical and histopathological features of 43 patients with DATC, evaluate their response to initial (surgery, ¹³¹I), and subsequent treatments (TKIs). A key objective was to analyze the disease-specific survival (DSS) in the entire cohort, with particular focus on differences between patients with locoregional disease and those with distant metastases.

## 2 Patients and methods

A consecutive series of 43 patients with DATC, who underwent surgery and were subsequently followed between 1998 and 2023, with a median follow-up of 10.1 years (IQR 4.0–16.3), were included. All patients were followed at the Endocrinology Thyroid Clinic, Garibaldi-Nesima Medical Center, Catania, Italy. Most patients underwent thyroidectomy ± lymph node dissection (27, 62.8%).

Histopathological variables (tumor size, histotype and variants, grading, extrathyroidal extension, number of lymph nodes examined, number and size of metastatic lymph nodes, location, and presence or absence of extranodal extension) were examined.

Tumors were staged according to the 8th TNM edition: T (maximum extent of the primary tumor) and N (regional lymph node metastases) were assessed at pathological examination, while M status (distant metastases) was assessed using the first post-surgical ¹³¹I whole-body scan (WBS) and/or additional diagnostic imaging.

At initial evaluation, patients were stratified into three risk categories (low, intermediate, high) according to the 2015 ATA risk classification system. Post-surgical RAI therapy was administered to patients according to current guidelines.

Patients were considered eligible if they had evidence of RAI-R, defined by meeting at least one of the following criteria: (1) presence of a measurable lesion without iodine uptake, (2) progression of at least one measurable lesion according to RECIST version 1.1 (11) within 12 months after RAI (radioiodine) therapy, (3) cumulative RAI activity exceeding 600 mCi, or advanced local disease not suitable for surgery.

Patient stratification into the ‘locoregional’ versus ‘distant metastatic’ groups was established based on radiological and anatomical criteria evaluated prior to TKI initiation. For patients presenting with *mixed disease* (the concurrent presence of both locoregional and distant lesions), adjudication was strictly determined by the specific disease compartment demonstrating objective radiological progression or critical anatomical compromise that represented the primary clinical indication for starting systemic therapy. The locoregional group included patients whose primary clinical threat and rationale for TKI treatment was symptomatic or rapidly progressive disease in the cervical region (e.g., impending airway compromise, invasion of the esophagus, or major vessels). Patients with mixed disease were adjudicated to this group if their distant metastases were radiologically stable, subclinical, or did not constitute the predominant compartment of objective disease progression. Conversely, the distant metastatic group comprised patients in whom the primary indication for TKI initiation was the progressive structural burden or symptomatic deterioration caused by distant lesions.

DATC patients with progressive or symptomatic disease started TKIs therapy. Systemic therapy was administered according to institutional multidisciplinary tumor board decisions. Regarding dose selection, individualized starting doses were frequently adopted. Since Lenvatinib is the most frequently used TKI and represents the first-line therapy, our analysis specifically addressed its dosing strategies and overall tolerability.

Treatment sequencing generally involved lenvatinib as the preferred first-line TKI, while other agents were reserved for subsequent lines or specific molecular indications. Regarding dose selection, while the standard recommended starting dose for lenvatinib is 24 mg/day, individualized starting doses were frequently adopted, for a subset of patients based on objective frailty criteria. This tailored dose-selection strategy was strictly intended to mitigate early severe toxicities and prevent premature, permanent treatment discontinuation.

All patients were followed periodically with serum thyroglobulin (Tg) and anti-Tg antibody measurements (AAT), neck ultrasound, and additional diagnostic imaging (computed tomography [CT], magnetic resonance imaging [MRI]). Locoregional treatments (e.g., radiotherapy, percutaneous ablation, surgical resection) were performed when clinically indicated.

Treatment efficacy was assessed according to RECIST version 1.1. Adverse events (AEs) were recorded and graded using the Common Terminology Criteria for Adverse Events (CTCAE) version 5.0 (12).

Genetic analysis was conducted through the application of next-generation sequencing (Ion Reporter, ThermoFisher) following standardized protocols. The sequencing panel encompassed a comprehensive set of genes (*BRAF*, *HRAS*, *KRAS*, *NRAS*, *TERT*, *TP53*, *PIK3CA*, *KMT2C*, *CDH4*, *ATM*, *CHEK2*, *ZFHX3*) and gene fusions/rearrangements (*RET*, *NTRK1/2/3*, *ALK*, *ROS1*, *PPARG*, *BRAF*, and *MET*) relevant to thyroid cancer pathogenesis (13). Genetic analysis is available in 17 patients undergoing therapy with TKI in the last 5–10 years, as per clinical practice since the biological samples of patients previously were not suitable for analysis.

Ethical approval was obtained from the institutional ethics committee. Written informed consent for publication was obtained from all patients.

## 3 Statistical analysis

Categorical variables were expressed as frequencies and percentages (%). Quantitative variables with a normal distribution were expressed as mean ± standard deviation (SD), while non-normally distributed variables were expressed as median with interquartile range (IQR). The normality of quantitative variables was tested using the Kolmogorov-Smirnov test. Categorical variables were analyzed using the Chi-square test with Yates’s correction or Fisher’s exact test. Kaplan-Meier (K-M) analysis was used to estimate overall survival (OS) and disease-specific survival (DSS), and the significance of differences was evaluated using the log-rank test.

A p-value <0.05 was considered statistically significant for all analyses. Data analysis was performed using Stata software, version 16 (StataCorp, College Station, TX, USA).

## 4 Results

### 4.1 Clinical and histopathological characteristics of DATC patients

The clinical characteristics of 43 DATC patients are shown in **Table 1a**. About half of the patients were female (22, 51.2%) with a F/M ratio of 1.0/0.9. The median age at diagnosis was 63.3 years (IQR 52.9–69.0).

**Table 1a:** Clinical and histopathological characteristics of 43 patients with advanced differentiated thyroid cancer.

Characteristics	n	%	Median (IQR)
Patients	43	—	—
Follow-up, years	—	—	10.1 (4.0–16.3)
Age, years	—	—	63.3 (52.9–69.0)
Gender (F/M, ratio)	22/21	—	1.0/0.9
ECOG Performance Status			
0–1	43	100	—
2	0	0	—
Histotypes			
Papillary	25	58.1	—
Follicular	10	23.3	—
Oncocytic carcinoma	1	2.3	—
Poorly differentiated	6	14.0	—
Papillary–follicular	1	2.3	—
Metastases at diagnosis (M1)	19	44.2	—
RAI therapy	36/43	83.7	—
RAI dose, mCi	—	—	287.5 (100–300)

ECOG, Eastern Cooperative Oncology Group; IQR,Interquartile range; RAI, Radioactive iodine. Percentages calculated over 43 patients unless otherwise specified.

Total thyroidectomy was performed in most patients (39, 90.7%), lobectomy alone in 1 patient (2.3%), and 3 patients (7.0%) initiated TKI therapy without prior surgery. For these three non-surgical patients, tumor histology and diagnosis were confirmed via biopsy of the primary thyroid tumor. Lymph node surgery was performed in 27 patients: 12 (27.9%) underwent central-compartment dissections and 15 (34.9%) combined central and lateral compartment dissections.

Among these patients, 25 (58.1%) had papillary carcinoma (PTC), 10 (23.3%) follicular carcinoma, 6 (14.0%) poorly differentiated carcinoma, 1 (2.3%) oncocytic carcinoma and 1 (2.3%) papillary–follicular carcinoma. Six patients (14.0%) had aggressive PTC variants, including tall cell, diffuse sclerosing, columnar, and insular. About half of the patients (19, 44.2%) had distant metastases at diagnosis or developed them after a short follow-up period.

Twenty-two patients (51.2%) had locally advanced disease, the majority of whom also had distant metastases (20/22, 90.9%). Regarding the extent of invasion within the 22-patient cohort, single-site involvement was identified in 45.5% (n=10) of cases, with the trachea being the most common isolated site (n=9) and one patient with major vessels involvement (4.5%). Multi-organ invasion was observed in the remaining 54.5% of the population, evenly distributed between double and triple-site involvement. Specifically, dual-site invasion occurred in 27.3% (n=6) of patients, involving combinations such as trachea-larynx (n=2), trachea-esophagus (n=2), trachea-major vessels (n=1) and mediastinum- major vessels (=1). Triple-site invasion was also recorded in 27.3% (n=6) of the cohort, with the concurrent involvement of the trachea, esophagus, and major vessels representing the most prevalent complex pattern (n=5) and one patient trachea, esophagus, and larynx. Overall, the trachea remained the primary focus of invasion, appearing in 90.9% of all analyzed cases”.

Post-surgery RAI therapy was administered to 36 patients (83.7%) with a median dose of 287.5 mCi (IQR 100–300 mCi). All patients had an ECOG performance status of 0–1 at the start of TKI therapy.

Among the 43 patients included in the analysis, the most prevalent comorbidity was hypertension, observed in 31 patients (72.1%), diabetes mellitus in 10 patients (23.3%), cardiomyopathy in 7 patients (16.3%), and chronic obstructive pulmonary disease (COPD) in 4 patients (9.3%) (**Table 1b**). These findings underscore the predominance of cardiovascular and metabolic comorbidities in the study cohort.

**Table 1b:** Comorbidities in 43 patients with advanced differentiated thyroid cancer.

*Comorbidity*	*No. of patients*	*%*
*Hypertension*	*31*	*72.1*
*Diabetes mellitus*	*10*	*23.3*
*BPCO*	*4*	*9.3*
*Cardiomyopathy*	*7*	*16.3*

Percentages calculated over 43 patients. BPCO = Bronchopneumopathy chronic obstructive.

A comparative baseline statistical analysis stratifying the overall cohort into two subgroups based on the presence of locoregional disease was carried out. The mean age at diagnosis was 59.1 ± 10.2 years (median: 62.5) for patients without locoregional involvement and 64.2 ± 14.3 years (median: 65.8) in the second group (p = 0.155). The gender distribution was balanced between the two cohorts (p = 0.763). Regarding the ECOG Performance Status and histological subtypes, no significant differences were observed (p = 0.240 and p = 0.473, respectively).

### 4.2 TNM staging system and risk stratification

The TNM classification and risk categories are presented in **Table 2**. According to the eighth edition of the TNM classification, the distribution of patients was as follows: 3 patients (7.0%) were classified as T1a, and another 3 patients (7.0%) as T1b; 8 patients (18.6%) as T2; 15 patients (34.9%), the largest group, were in the T3a category; 6 patients (14.0%) were T3b; 2 patients (4.7%) were T4a; and finally, 6 patients (14.0%) were classified as Tx. Regarding nodal status, 16 patients (37.2%) were classified as Nx; 7 patients (16.3%) had negative regional lymph nodes (N0); 5 patients (11.6%) were classified as N1a and 15 patients (34.9%) as N1b.

**Table 2:** TNM staging (8th edition) and risk categories (2015 ATA guidelines) distributions in 43 patients with DATC.

	n	%
TNM (8th edition)		
T status		
T1a	3	7.0
T1b	3	7.0
T2	8	18.6
T3a	15	34.9
T3b	6	14.0
T4a	2	4.7
Tx	6	14.0
N status		
Nx	16	37.2
N0	7	16.3
N1a	5	11.6
N1b	15	34.9
M1	19	44.2%
Staging (8th edition)		
<55 years		
Stage I	8	18.6
Stage II	4	9.3
≥55 years		
Stage I	2	4.7
Stage II	12	27.9
Stage III	0	0.0
Stage IVA	9	20.9
Stage IVB	8	18.6
Risk categories (2015 ATA guidelines)		
Low	11	25.6
Intermediate	9	20.9
High	23	53.5

TNM, Tumor–Node–Metastasis; ATA, American Thyroid Association; Percentages calculated over 43 patients.

Among patients younger than 55 years, 8 (18.6%) were stage I and 4 (9.3%) stage II. For patients aged ≥55 years, 2 (4.6%) were stage I, 12 (28.0%) stage II, 9 (20.9%) stage IVA, and 8 (18.6%) stage IVB.

According to the 2015 ATA risk categories, the distribution of patients was as follows: 11 patients (25.6%) were classified as low risk, 9 (20.9%) as intermediate risk, and 23 (53.5%) as high risk.

### 4.3 Therapy with tyrosine kinase inhibitors and outcomes

The median interval between diagnosis and the onset of radioiodine refractoriness was 5.9 years (IQR: 2.6–12.2).

The median age at initiation of TKIs therapy was 71 years (IQR: 62.2–75.5).

First line therapy was Lenvatinib in 40 patients, Vandetanib in 2 and Sorafenib in 1.

During follow-up, 10 patients required treatment modification due to disease progression or adverse events: two initiated Selpercatinib (one in combination with radiotherapy), two Cabozantinib, and one Dabrafenib plus Trametinib; in addition, three underwent radiotherapy, two chemotherapy, and one resumed radioiodine therapy.

At the beginning of the treatment, nearly all patients (41/43) presented with distant metastases (15 lung, 2 bone, 16 lung and bone, 3 liver and lung, and 5 multiple sites [>2]).

The median initial Lenvatinib dose was 14 mg (range, 4–24 mg). Among the 43 patients, 32.5% started at 24 mg, 7.0% at 20 mg, 16.3% at 14 mg, 16.3% at 10 mg, and 27.9% at 4 mg. The median time to dose reduction was 1.7 months (0.8–3.5), and the median time to best response was 4.6 months (3.4–9.0).

At last follow-up, 22 patients (51.2%) had stable disease and 5 (11.6%) showed disease progression. No major locoregional complications were observed during TKI therapy.

Overall, 27 patients (62.8%) were alive and 16 (37.2%) had died from the disease (**Table 3**). Among these, 7 deaths were primarily attributable to locoregional progression, while 7 deaths were related to the progression of distant metastatic disease and 2 to both sites of progression (local and distant disease).

**Table 3:** Status of disease at initiation of systemic therapy, dose reduction, and outcomes in 43 patients with DATC.

Variable	n./tot pts	%	Median (IQR)
Radioactive iodine-refractory (from diagnosis)		—	5.9 years (2.6–12.2)
Metastatic sites			
Lung	15/43	34.9	—
Bone	2/43	4.6	—
Lung + Bone	16/43	37.2	—
Liver + Lung	3/43	7.0	—
Multiple (>2 sites)	5/43	11.6	—
Locally advanced disease	22/43	51.2	—
Age at TKIs initiation		—	71 years (62.2–75.5)
Lenvatinib first dose (mg)			14 (4–24)
24 mg	14/43	32.5	—
20 mg	3/43	7.0	—
14 mg	7/43	16.3	—
10 mg	7/43	16.3	—
4 mg	12/43	27.9	—
Time to therapy reduction		—	1.7 months (0.8–3.5)
Best response to Lenvatinib		—	4.6 months (3.4–9.0)
Status at last visit			
Progression	5/43	11.6	—
Stable disease	22/43	51.2	—
Survival status			
Alive	27/43	62.8	—
Dead (disease-specific)	16/43	37.2	—
Median time to death (from Lenvatinib start)	16	—	22.2 months (5.0–33.0)

IQR, Interquartile range; DATC, Differentiated advanced thyroid carcinoma; Percentages are calculated over 43 patients, unless otherwise specified.

### 4.4 Molecular analyses

Comprehensive molecular profiling was performed on a cohort of 18 patients (details in **Table 4**). Molecular profiling revealed a markedly heterogeneous mutational landscape. The most frequently observed alterations was BRAF in 7 patients (isolated or in combinations) and RAS in 6 patients (3 NRAS, 2 HRAS and 1 KRAS). All other variants occurred only once across the cohort, emphasizing the high degree of inter-individual genomic heterogeneity. Evaluation of *TERT* promoter status was available for three cases, revealing one mutated tumor and two wild-type cases.

**Table 4:** Molecular markers in 18 patients.

N. pts	Molecular markers
7	BRAF v600e *
6	RAS **
2	RET (NCOA1-RET, CCDC6)
1	TP53 Ser183Ter
1	ZFHX3
1	None

*(3 isolated, 1 with *KMT2C* and *CDH4*, 1 with *TP53*, *TERT* and *PIK3CA*, 1 with *RET* and *TP53* and *KMT2C*, 1 with *TP53* and *TERT*); **(2 *HRAS*, 2 *NRAS*, 1 *KRAS*, 1 *NRAS* with *MET*).

Among the seven patients harboring *BRAF* mutations, five (71.4%) presented with predominantly locoregional disease, whereas among those with *RAS* mutations, only two (33%) showed disease confined to the locoregional compartment.

Comparing the distribution of locoregional versus distant disease between patients with *BRAF* and *RAS* mutations, there was no statistically significant difference between the two groups (p = 0.3), a result likely influenced by the small sample size. Nevertheless, a trend emerged suggesting that *BRAF*-mutated tumors were more frequently associated with locoregional involvement, whereas *RAS*-mutated tumors appeared more commonly linked to distant spread.

### 4.5 Survival analysis

**Figure 1:**
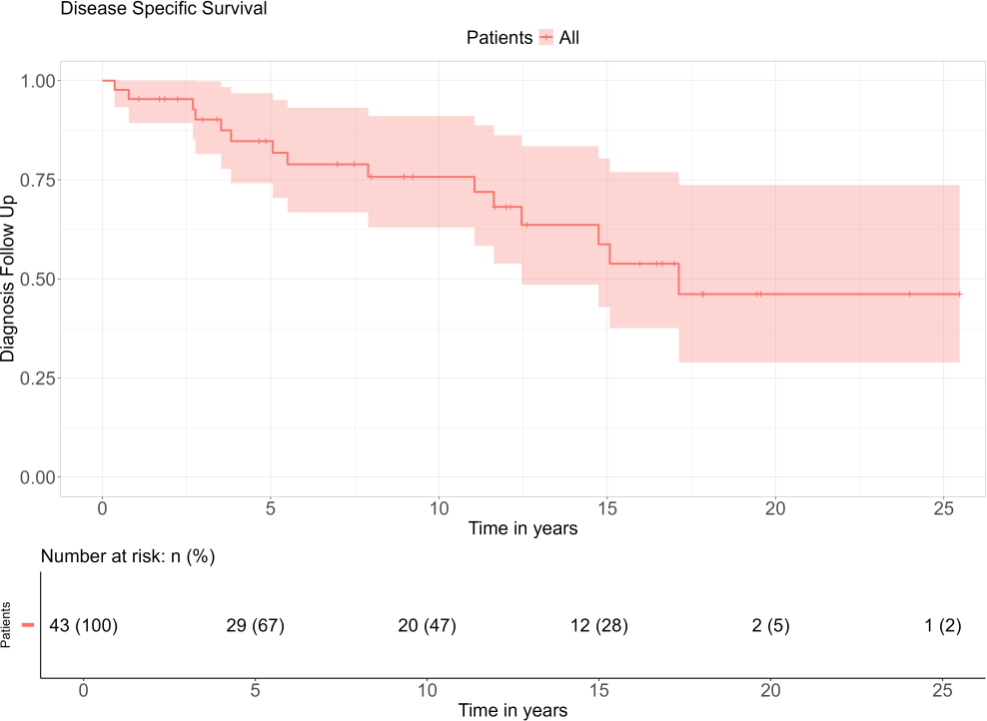
Kaplan-Meier curve showing disease-specific survival (DSS) from the time of diagnosis in 43 patients with DATC. Shaded areas represent 95% confidence intervals. At-risk tables below the graph report the number of patients still under observation at each time point (percentages in brackets). Median DSS was 17.1 years (95% CI: 12.5-NA), with estimated survival rates of 84.7% at 5 years, 75.7% at 10 years, 58.7% at 15 years, and 46.1% at 20 and 25 years.

The disease-specific survival from the time of diagnosis was analyzed in 43 patients (**Figure 1**). A total of 16 disease-related deaths were recorded. The median DSS was 17.1 years (95% CI: 12.5–NA). Estimated survival rates were 84.7% at 5 years (95% CI: 74.1–96.8), 75.7% at 10 years (95% CI: 63.0–91.1), 58.7% at 15 years (95% CI: 42.9–80.3), and 46.1% at both 20 and 25 years (95% CI: 28.9–73.7). The curve indicates favorable short- and mid-term outcomes, with more than 80% of patients surviving at 5 years. However, survival progressively declined over time, reaching less than 50% beyond 20 years. The widening confidence intervals in the later follow-up reflect the small number of patients at risk.

**Figure 2:**
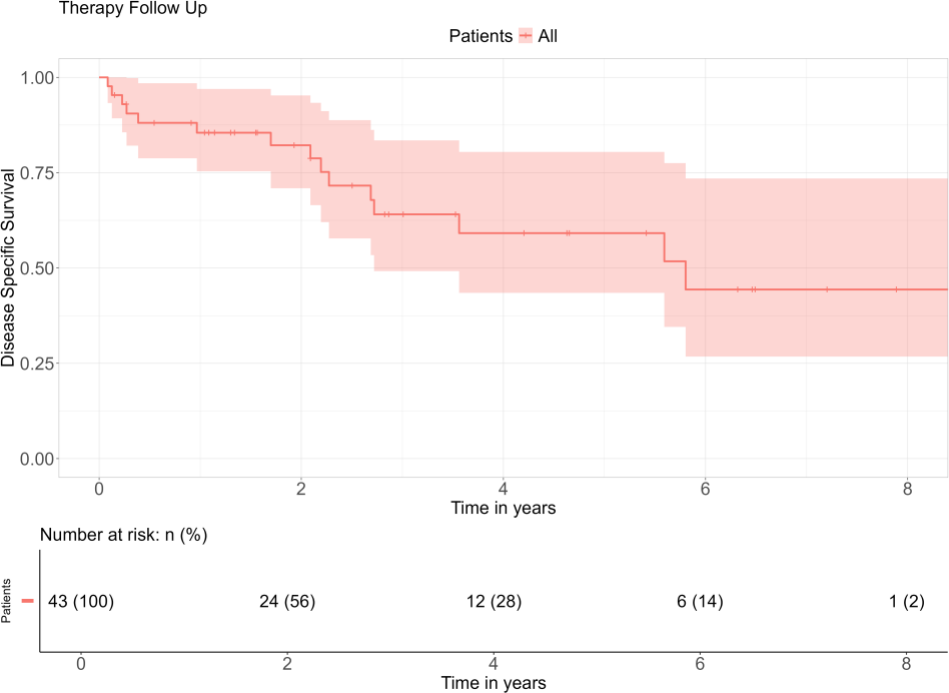
Kaplan-Meier curve of disease-specific survival (DSS) from the start of systemic therapy in 43 patients with advanced differentiated thyroid cancer. Shaded areas represent 95% confidence intervals. The median DSS was 5.8 years (95% CI: 2.7-NA). At 1, 2, and 5 years, DSS rates were 85.5%, 82.2%, and 59.1%, respectively.

From the start of systemic therapy, the DSS of the entire cohort (n = 43) was 85.5% (95% CI: 75.4–97.0) at 1 year, 82.2% (95% CI: 70.9–95.3) at 2 years, and 59.1% (95% CI: 43.5–80.4) at 5 years (**Figure 2**). The median DSS was 5.8 years (95% CI: 2.7–NA). A gradual decline in survival was observed after the second year of treatment, with fewer than half of the patients alive beyond 6 years of follow-up.

**Figure 3:**
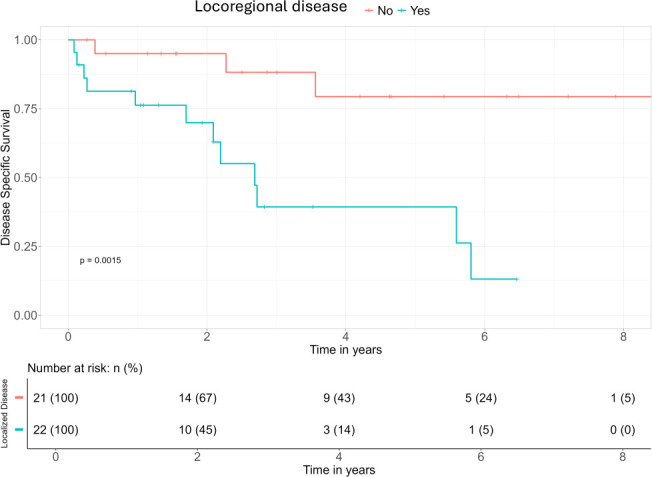
Kaplan-Meier curves for disease-specific survival (DSS) according to the presence of localized disease. The difference between groups was statistically significant (log-rank test, p = 0.0015).

DSS differed significantly according to the presence of locoregional disease (**Figure 3**). Patients without locoregional disease (n = 21) showed a DSS of 95.0% (95% CI: 85.9–100) at both 1 and 2 years, and 79.4% (95% CI: 60.5–100) at 5 years. In contrast in the locoregional group, survival rates were 77% at 1 year, 59% at 2 years, and 27% at 5 years with a median survival was 2.7 years (95% CI 2.1–NA). In univariate analysis, the presence of locoregional disease was associated with a more than six-fold increased risk of DSS mortality (Hazard Ratio, HR, of 6.21, 95% CI 1.73–22.3; p = 0.005).

## 5 Discussion

Thyroid cancer is the most frequent neoplasm of the endocrine system (14), accounting for approximately 90% of endocrine tumors. More than 85% of differentiated thyroid carcinoma (DTC) cases exhibit a papillary histotype. The estimated 5-year overall survival rate for thyroid cancer is 98.1%, with 99.9% for locoregional disease and 55.5% for distant metastases. Although DTCs generally have a favorable prognosis, a subset of cases display aggressive tumor behavior and poor clinical outcomes (5).

Advanced thyroid carcinoma (DATC) represents a rare condition, comprising approximately 5% of all thyroid cancers, with an incidence of 4–5 cases per 1,000,000. DATC includes radioiodine-refractory thyroid carcinomas (RAI-R-TC) and tumors not amenable to thyroidectomy, for which treatment with radioactive iodine is not indicated.

Regarding the clinical and histopathological characteristics of DATC, in our study, which included 43 patients, there was an almost equal sex distribution with a median age at diagnosis of 63.3 years and at initiation of TKIs therapy was 71 years. The majority of tumors were papillary (58.1%), followed by follicular (23.3%) and poorly differentiated (14%) ones. Nineteen patients (44%) were metastatic at diagnosis. Our findings align with previous studies. In real world data (15) the median age at enrollment was 60 years and half of the patients were females; in this series all patients had metastatic disease with progression of disease within the last 12 months. The Austrian cohort (16) comprised 43 patients with a median age of 70 years, the majority of tumors were of the papillary subtype (86%). The cohort described by Porcelli et al. (n=23) included 14 females and 9 males, with a median age at diagnosis of 62 years (IQR: 53.5–65) and at lenvatinib initiation of 68 years (IQR: 61–74). In that study, 9 patients (39%) presented with metastases at diagnosis. In the real-world study by Worden et al. (18) 308 patients were included: 51.6% female, median age at starting TKI was 60 years old and there was the same distribution between papillary and follicular histotypes.

The molecular heterogeneity observed in our cohort is consistent with evidence reported in recent genomic studies of advanced and radioiodine-refractory thyroid cancer. As seen in larger series, *BRAF* and *RAS* mutations represented the most recurrent alterations, although their prevalence in small cohorts often fluctuates widely depending on patient selection and disease stage. The limited identification of *TERT* promoter mutations in our series—with 1 mutated and 2 wild-type cases—is likely due to testing older primary tumor samples rather than metastatic tissue, thereby missing alterations that typically arise later during tumor dedifferentiation and progression.

In the literature, while individual mutation rates vary widely depending on the degree of dedifferentiation (*TERT* 40-75%, *TP53* 10-79%, *PIK3CA* and *KMT2C* 10-20%), the presence of co-mutations involving these specific genes has been linked to aggressive clinical behavior and poorer outcomes (17).

Indeed, recent literature increasingly highlights the critical clinical utility of BRAF and TERT promoter mutations in the precision management and risk stratification of papillary thyroid carcinoma (18, 19). Furthermore, ongoing regional pilot studies emphasize the importance of evaluating the prevalence and prognostic weight of the BRAF V600E mutation across diverse populations to tailor individualized treatments (20).

In our cohort, 36 patients (83.7%) received RAI therapy, with a median cumulative dose of 287.5 mCi (IQR: 100–300), which was lower than in Porcelli et al.’s series (21) (median 410 mCi, IQR: 300–670; 22/23 patients treated). Three patients in our study had received another TKI prior to lenvatinib: one (2.3%) sorafenib and two (4.6%) vandetanib. In Locati et al.’s (15) larger series of 94 patients with a median of enrollment was 60 years and 49% female, nearly all patients (98%) underwent primary surgery and RAI therapy. Before lenvatinib, 64% had received sorafenib, 22% chemotherapy, 18.3% vandetanib, 8.3% sunitinib, and 13.3% other systemic treatments.

Comorbidities were common: in Locati et al.’s study (15), 60% of patients had hypertension and 14% diabetes, whereas in our series 72.5% had hypertension and 23.3% diabetes. Our data revealed a notable pattern of multisite metastatic involvement, with 55.8% of patients presenting with two or more sites. These observations are consistent with those reported in the Austrian real-world cohort (16): metastases to the lymph nodes (74%), lungs (86%), bone (35%), liver (16%), and brain (12%). In Worden study (22) about 40% of patients had lymph node metastases, 33% lung, 20% bone and 15% liver metastases.

Regarding treatment duration and dosing, our study revealed a median treatment duration with Lenvatinib of 25 months (2.1 yrs), similar to that in Austrian real world data (27.6 months), longer than 17.5 months reported in Worden study and about 6 months reported in real world (15, 22). In our study, the starting dose of lenvatinib differed significantly from other studies. In our cohort, the median starting dose was 14 mg; specifically, 15 patients (34.9%) of patients started at 24 mg, 12 (27.9%) at 4 mg and an additional 14 patients received initial doses of 10 or 14 mg, due to locally advanced tumor or severe comorbidities. Conversely, in literature most patients (about 70%) started with 24 mg/day (15, 21, 22). Moreover in Locati study (15), dosing was reduced in 62 patients, with a median reduction of 41.7% from baseline. In the Austrian cohort (16), all patients initiated lenvatinib treatment at the standard starting dose of 24 mg daily. During therapy, dose reductions were required in the majority of patients, resulting in a median maintenance dose of 14 mg/day and a notable proportion of patients (91%) necessitated lenvatinib dose reductions during the course of treatment. In Worden study (22), 62% of patients initiated lenvatinib at 24 mg and only 8% required dose modifications (decrease, increase or interruption). In our practice, a more conservative dosing strategy was adopted to minimize severe or intolerable adverse events, thereby improving patient compliance and reducing the likelihood of treatment interruptions or dose reductions.

The median time to best response was similar in our study (4.6 months) compared with data in literature (21). In Austrian real world biochemical response markers indicated an early therapeutic effect: serum Tg levels decreased by approximately 75% after one month and by up to 98% after three months of treatment, suggesting a rapid biochemical response to lenvatinib.

At last follow-up, 22 patients were stable, whereas 20 had progressed or died from the disease; 27 patients remained alive. In Porcelli et al.’s study (21), 14 (60.9%) patients were alive and 9 had died, with 13 of the survivors stable and only one in progression.

In the present series, DSS appears broadly favorable when compared with both pivotal and real-world lenvatinib cohorts. The median DSS of 17.1 years and 5-year DSS of 84.7% from diagnosis indicate excellent overall disease control in the long term. When survival was calculated from the start of systemic therapy, DSS rates of 85.5% at 1 year, 82.2% at 2 years, and 59.1% at 5 years were observed, with a median DSS of 5.8 years. These findings are consistent with, and in some respects exceed, those reported in the Austrian cohort (16), where 2-year and 5-year OS were 74% and 59%, respectively, and the median treatment duration was 27.6 months.

Compared with the SELECT phase III trial, which demonstrated a median PFS of 18.3 months, the current data suggest that real-world lenvatinib use may yield prolonged survival in selected patients, particularly when maintained therapy is feasible over several years.

Relative to other real-world reports, such as Locati et al. (15) (median OS 23.8 months, PFS 10.8 months), the present cohort displays substantially longer survival. Notably, in our cohort, a significant prognostic distinction was observed between patients with and without locoregional disease at therapy initiation: the sixfold higher mortality risk in the locoregional group (HR 6.21, 95% CI 1.73–22.3, p = 0.005) underscores the critical prognostic influence of disease extent.

Furthermore, our findings align with the results reported by Lamartina et al. (23) in their analysis of 22 patients with unresectable DTC. They observed that local progression was a primary driver of mortality, accounting for 7 out of 11 disease-related deaths. This supports our observation that advanced locoregional disease remains a critical determinant of survival, even in the presence of distant metastases. In Worden real world (22) the reported PFS was 49 months; 75% of patients were alive at last follow-up (OS 90.8% at 12 months, 78.4% at 24 months and 57% at 72 months).

The observed difference in DSS between patients with locoregional and distant metastatic thyroid cancer underscores the heterogeneity of prognosis within the same tumor type. These findings suggest that a uniform treatment approach may not be adequate. Instead, therapeutic strategies should be differentiated and tailored to the extent of disease at diagnosis, with more aggressive interventions or novel therapeutic approaches warranted in patients with poor-prognosis subgroups.

A multidisciplinary approach is essential for managing high-risk locoregional disease, in accordance with recent ESMO and ATA clinical practice guidelines for thyroid cancer (24). This includes integrating EBRT which remains a cornerstone for locoregional control, particularly in unresectable disease, neoadjuvant TKI therapy to enable surgical resection through tumor shrinkage. Furthermore, the therapeutic landscape is rapidly evolving to include newer molecular-targeted therapies and immunotherapy combinations (e.g., lenvatinib combined with pembrolizumab). These novel immunotherapeutic strategies are currently under active investigation to overcome TKI resistance and improve long-term outcomes in advanced disease (25).

Although distant metastases are classically associated with poor prognosis in many cancers also in DATC, there is emerging evidence as in head and neck and esophageal malignancies that very advanced locoregional disease, particularly when key structures are involved (e.g. tracheal, esophageal, or major vascular involvement), had worse prognosis than that of patients presenting with distant metastases only. However, to our knowledge there has been no systematic comparison quantifying the difference in outcome between invasive locoregional disease and distant metastatic disease in advanced RAI-R TC. Most studies enroll mixed populations and report outcomes by histology, age, prior RAI, tumor burden, or mutational status, rather than by anatomical site of dominant disease.

According to the EHNS–ESMO–ESTRO guidelines (26), locally advanced squamous cell carcinomas of the larynx or hypopharynx often require multimodal therapy, yet long-term outcomes remain poor. Five-year overall survival for unresectable T4 laryngeal cancers rarely exceeds 30–35%, while hypopharyngeal cancers show 5-year survival rates of only 20–35% despite aggressive treatment. Similarly, locally advanced esophageal carcinoma, even in the absence of distant spread, carries a median survival of approximately 12–18 months with definitive chemo-radiotherapy, and 5-year survival of 15–25% (27). Primary tracheal tumors, although rare, follow a comparable pattern, with unresectable disease associated with median survival of less than one year and resected cases reaching 5-year overall survival of only 30–40% (28). These examples underscore that across different head and neck sites, locoregional extension into critical structures such as the trachea, esophagus, or major vessels significantly worsens prognosis and often dictates therapeutic strategy, sometimes with outcomes as unfavorable as those seen with distant metastases.

The present study is characterized by some limitations, primarily its single-center nature and the cohort size of 43 patients. Although a small cohort in absolute statistical terms, since advanced thyroid carcinoma receiving tyrosine kinase inhibitor therapy is a rare disease, our cohort of 43 patients represents a substantial sample for a single-center study. Specifically, it restricted our ability to perform broad subgroup analyses and precluded the use of multivariate survival models (e.g., Cox proportional hazards regression) to adjust for potential confounders, and warrants caution when generalizing these conclusions. Another limitation is the intrinsic heterogeneity of TKI regimens within our real-world cohort, including the relatively low starting dose of lenvatinib compared to pivotal trials. In our real-world practice, reduced doses were tailored to manage patient frailty and mitigate early toxicities, preventing permanent treatment discontinuation While this tailored approach is clinically justified to maintain treatment adherence, we acknowledge that such treatment heterogeneity introduces a potential confounding bias in our DSS comparisons. Although dose intensity is a known prognostic factor, our limited overall sample size precluded a statistically robust sub-analysis correlating the specific initial dose with survival outcomes.

## 6 Conclusions

In conclusion, our preliminary data suggest a marked difference in disease-specific survival between patients with locoregional and distant metastatic thyroid cancer highlights the heterogeneity of prognosis within this tumor entity. Despite belonging to the same histological group, the survival trajectories of these patients appear profoundly divergent, suggesting that a uniform therapeutic approach may be insufficient. These findings underscore the need for distinct and personalized therapeutic strategies to improve survival across different patient subgroups. These tumors pose a significant clinical challenge, often characterized by adverse prognosis and an incomplete biological understanding.

## Data Availability

The raw data supporting the conclusions of this article will be made available by the authors, without undue reservation.
